# A Pharmacist-Led Quality Improvement Project to Optimize Medication Evaluation and Reconciliation in Home Healthcare

**DOI:** 10.1097/NHH.0000000000001377

**Published:** 2025-09-08

**Authors:** Jeffrey A. Clark, Kimberly C. McKeirnan, Brian J. Gates

**Affiliations:** **Jeffrey A. Clark, PharmD, BCGP,** is an Associate Professor, Department of Pharmacotherapy, College of Pharmacy and Pharmaceutical Sciences, Washington State University, Spokane, Washington.∗; **Kimberly C. McKeirnan, PharmD, BCACP,** is a Professor, Department of Pharmacotherapy, College of Pharmacy and Pharmaceutical Sciences, Washington State University, Spokane, Washington.; **Brian J. Gates, PharmD, BCGP,** is a Professor, Department of Pharmacotherapy, College of Pharmacy and Pharmaceutical Sciences, Washington State University, Spokane, Washington.∗

## Abstract

Medication reconciliation was adopted as a National Patient Safety Goal by the Joint Commission in 2005 and is now standard practice across care settings. More recently, the concept of medication optimization has gained attention, recognizing that safe medication use requires more than reconciliation alone. Home healthcare (HHC) is one setting with a critical need for medication optimization. This work describes a pharmacist-led interdisciplinary team (IDT) effort to reduce hospitalization rates at Providence VNA Home Health by improving medication reconciliation, evaluation, and prescriber communication. The IDT developed a tool and a 1-hour training with operational definitions and scenarios for reconciliation and documentation, along with a separate training focused on medication evaluation. To assess training effectiveness, the primary outcome was to reduce 30-day hospitalizations among high-risk heart failure patients to below 12%. This outcome was met and sustained for 8 weeks post-implementation. A secondary goal—reducing 30-day rehospitalizations per Strategic Healthcare Programs (SHP)—was also met and sustained from April to December 2020. This quality improvement project demonstrated that enhancing medication reconciliation and evaluation in high-risk patients reduces hospitalizations. Reconciliation may be especially important in patients with two or more self-reported unreconciled medications in the EHR, which may signal suboptimal medication evaluation. Addressing the challenges HHC clinicians face in optimizing medications and reinforcing best practices can improve outcomes. Pharmacists play a key role in interdisciplinary teams in HHC, given the complexity of medications and their impact on quality measures.

**Figure:**
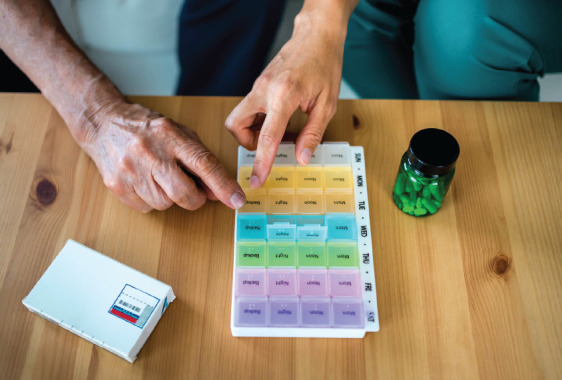
No caption available.

Home healthcare (HHC) agencies are required to complete a medication regimen review (MRR) for patients as part of the comprehensive assessment, and MRRs are a standard of care across multiple settings. Medication reconciliation alone will not ensure that medications are safe and effective for patients nor satisfy all the required components of the MRR. It was hypothesized that failure to optimize medications and address medication-related problems (MRPs) through adequate medication reconciliation and evaluation by HHC clinicians may lead to hospitalizations. As a result, a quality improvement (QI) team, including the onsite academic consultant pharmacy team, was convened as part of a grant to undertake a project with the following objective: to reduce hospitalizations by optimizing medication reconciliation; medication evaluation; and prescriber communication through a collaborative, interdisciplinary approach, at a HHC agency.

At the time of the project, Providence VNA Home Health (Providence VNA) was a nonprofit HHC agency with approximately 3,500 admissions per year. This agency provided a variety of skilled services offered by nurses, physical therapists, occupational therapists, speech-language pathologists, medical social workers, and home health aides. Beginning in 1994 Providence VNA Home Health had a unique relationship with the Washington State University College of Pharmacy and Pharmaceutical Sciences (WSUCPPS) to have a pharmacy consulting service onsite (Providence VNA Home Health, n.d.; Washington State University College of Pharmacy and Pharmaceutical Sciences, n.d.). The partnership with WSUCPPS was established based on the recognition that HHC agencies deal with complex medication-related issues and the belief that an onsite pharmacy presence could help to address MRPs. Faculty, Doctor of Pharmacy (PharmD) residents, and PharmD candidates from WSUCPPS offered two primary services, a referral service, and a high-risk service. The referral service allowed HHC clinicians to request pharmacist assistance to address any potential medication-related issues (Clark et al., 2019). The high-risk service focused on the reduction of hospitalizations in patients considered to be at high risk for hospitalization as defined in Table 1. The pharmacy team began screening high-risk patients upon admission to HHC in 2015 for MRPs, primarily with a focus on issues beyond reconciliation. In addition to clinical services, the pharmacy team provided medication-related education to the clinicians at the site.

**Table 1:** Patients at High-Risk for Hospitalization

Primary ICD-10 diagnosis code for home healthcare containing the following:	Heart failure COPD Recent myocardial infarction Pneumonia Respiratory failure and one of the above diagnoses on the patient's problem list
Free text home healthcare episode name including:	“heart failure” “CHF” “COPD” “Myocardial infarction” or “NSTEMI” or “STEMI”

## Background

Medication reconciliation performed at a level to meet federal regulations has grown in complexity (Innes et al., 2024; Medicare and Medicaid Program: Conditions of Participation for Home Health Agencies, 82 Fed. Reg. 9, 2017). The end product of medication reconciliation using the best possible medication history (BPMH) should clearly communicate in the electronic health record (EHR) both what the patient is taking and what is currently ordered (Almanasreh et al., 2016). However, it is not unusual for HHC agencies to fall far short of accomplishing this task, which may lead to significant patient harm, including hospitalization, especially when poor medication reconciliation is coupled with or causes poor medication evaluation (Brody et al., 2016; Coleman et al., 2005; Corbett et al., 2010; Fuji & Abbott, 2014; Michel et al., 2013; Mixon et al., 2020; Neumiller et al., 2017). Pharmacist interventions to resolve MRPs either directly or on an interdisciplinary team (IDT) have been shown to be effective in multiple settings, including HHC (Clark et al., 2016; Koshman et al., 2008; Mckay et al., 2019; Parajuli et al., 2019; Reidt et al., 2014; Roughead et al., 2009). Collaboration between pharmacists and nurses may be most important for patients with heart failure (HF; Schumacher et al., 2021). The literature demonstrates a broad range of medication issues in HF patients that could benefit from resolution (Foust et al., 2012; Koshman et al., 2008; Mckay et al., 2019; Parajuli et al., 2019; Roughead et al., 2009; Schumacher et al., 2021; Triller & Hamilton, 2007). Many of these issues are not directly related to HF. Only 35.2% of patients with HF are readmitted for HF (Dharmarajan et al., 2013). Similarly, another study showed that for HF patients only 22.5% of total MRPs are due to HF medications (Gastelurrutia et al., 2011). Additionally, timely intervention is important as 60% of readmissions in patients with HF occur within 15 days of hospital discharge (Dharmarajan et al., 2013).

This work describes a QI project undertaken by a pharmacist-led IDT to reduce hospitalizations at Providence VNA Home Health by improving the processes surrounding nurse and therapist medication reconciliation and evaluation. An in-depth review of the above literature may be found in the supplement at http://links.lww.com/HHN/A170.

## Methods

### Design

The pharmacy faculty members based at Providence VNA, along with another pharmacy colleague from WSUCPPS, received a grant for completion of improvement coach training (completed during project Weeks 1-15) and participation in a learning action network (LAN; Weeks 25-58) both led by faculty with the Institute for Healthcare Improvement (IHI, n.d.). This QI project was conceived during the improvement coach training, executed during the LAN, and monitored following the LAN. With this project, an IDT was convened in fall 2018 to identify ways to optimize medications in home health. All other members of the IDT were from Providence VNA, including one field nurse, one physical therapist, one nurse unit manager, and one administrator. In reference to specific weeks mentioned throughout the paper, Week 1 was the start of the improvement coach training since the training and project are linked together. The final change package was rolled out to the whole agency in Weeks 56 and 57. Patient level data evaluation ended in Week 66, whereas aggregate hospitalization data were monitored monthly in SHP through Week 127.

The IDT initially focused on meeting with the five different geographically based care teams at the site to gather information about key issues to the clinicians, which led to development of a fishbone diagram to identify potential drivers of unreconciled medications (Supplement Figure S1 at http://links.lww.com/HHN/A176) and a block diagram showing the steps involved in medication reconciliation and evaluation as well as the barriers to each step (Supplement Figure S2 at http://links.lww.com/HHN/A177). Based on this process, there was a clear need for further training regarding the best practices for using the EHR for medication reconciliation and evaluation. To determine further where to focus, five patients and five HHC clinicians were interviewed (Supplemental Table S1 at http://links.lww.com/HHN/A171). The IDT determined that the primary process measure should focus on the number of unreconciled medications based on brief review of the medication list after the SOC because this is a simple way to see if the entire process of reconciliation and evaluation was being followed.

**Figure 1.**
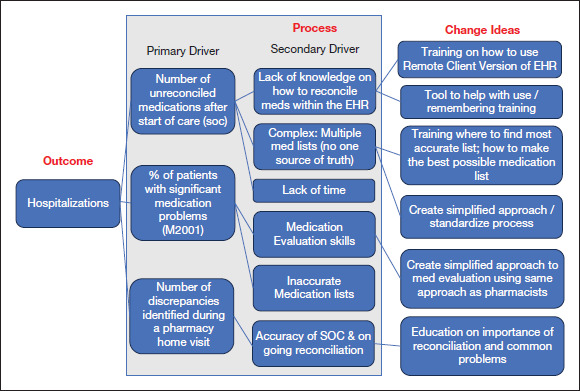
Expanded Driver Diagram.

In acknowledging that one of the barriers to adequate medication reconciliation and evaluation is the time required, the IDT wanted to design the QI project around outcomes that clinicians would deeply engage with and chose hospitalizations. From the fishbone and block diagrams a driver diagram was developed to help identify primary and secondary drivers of hospitalizations (see Figure 1). The IDT selected heart failure (HF) hospitalization rates as the primary outcome measure based on the previously mentioned literature regarding heart failure and medications. As secondary outcomes, the hospitalization rates for the broader categories of patients deemed as high risk for rehospitalization and overall hospitalization rates for all patients at the agency were also evaluated. The specifics targets of these outcomes were the following:

- Primary Outcome Measure: To reduce 30-day hospitalization in high-risk patients with HF from an average of 22.5% (median 21.1%) down to 12% by project Week 66
- Secondary Outcome Measure 1: To reduce 30-day hospitalization in all high-risk patients from an average of 20.45% (median 19.4%) to 12% by project Week 66
- Secondary Outcome Measure 2: To reduce 30-day rehospitalization rate in all patients from a median of 12.1% to a median of 9% (OR target 75th percentile) by project Week 66

To identify high-risk patients, the pharmacy team's high-risk service definition of patients at high risk for hospitalization was used (see Table 1). The key outcome, process, and balancing measures are described in Supplemental Table S2 at http://links.lww.com/HHN/A172. The reconciliation process measures were completed via the EHR. In the EHR, medications appear in two sections: “on the plan of care” and “off the plan of care.” Unreconciled medications were defined as medications in the EHR that were “off the plan of care” but were not marked as “not taking.” Per the processes at the site and developed as part of this project, no medications remained on the “off of the plan of care” section unless they could not be removed by the HHC clinicians at SOC. When medications could not be removed (because they could only be removed by the prescriber) they were marked as “not taking” while waiting for the prescriber to remove the inactive medication from the list. Following this process clearly communicated to other providers using the EHR that the patient was not taking the medication, which had been an identified source of confusion as all orders appeared active regardless of being on or off the HHC agency's POC.

As a balancing measure to ensure the change package wasn't adversely affecting other aspects of patient care, clinicians who had completed the training were asked to respond to a one-question anonymous poll inviting them to report whether the new process took more, less, or about the same amount of time. The total time required for medication optimization was identified as a potential barrier early in project development and therefore was assessed.

During the project, outcomes were stratified based on how medications were listed in the EHR, which is either “entered by a prescriber” or as “patient-reported.” Those entered by a prescriber were from prescribers in the same health system as the HHC agency, whereas patient-reported medications could refer to any medication entered by someone who was not an agent of the prescriber. If an HHC clinician entered a medication in the home, it would appear as “patient-reported.” Outcomes were also stratified into two groups based on the process measure of the number of unreconciled medications (either one or no unreconciled medications vs two or more). This separation was made based on an initial analysis before the rollout of interventions to the whole agency indicating a potential difference in these groups.

The interventions were developed in a reiterative manner and included both comprehensive training on using the EHR for different scenarios for medication reconciliation and a separate training specifically on medication evaluation.

The local Providence Institutional Review Board determined this project is QI. The SQUIRE 2.0 Revised Standards for Quality Improvement Reporting Excellence were reviewed in the reporting of this work (SQUIRE, n.d.).

### Strategy

Following IHI training and initial planning, the first PDSA (plan, do, study, act) cycle occurred in Week 31 (see Table 2 for full project timeline and list of PDSA cycles) to assess the feasibility of initial ideas for a medication reconciliation tool. During the pilot over several weeks, clinicians from one care team used a playground environment in the EHR and the tool during live, in-person trainings. They worked through scenarios developed by the IDT that required specific actions—or combinations of actions—in the EHR to effectively update and communicate changes. The tool, reconciliation training scenarios, and delivery all continued to be refined through PDSA cycles with the valley team. A preliminary analysis of the impact on medication reconciliation and hospitalizations on the valley team showed improvement which coincided with Week 37 and supported moving forward with scaling the intervention to the full agency. Ultimately, from these small tests of change, 13 different reconciliation scenarios were identified and re-created on a single patient in a playground environment for the EHR. Based on feedback related to different learning styles, a tip sheet was developed that summarized the key steps for reconciliation in a different format and layout.

**Table 2.** Overall Project Timeline and Plan Do Study Act (PDSA) Cycles

Week	
1-15	**Improvement Coach Training**
1-30	**IDT team formation, initial meetings, literature review, creation of charter, earliest baseline measurements, identification of specific aims**
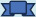	**Small scale tests of change**
**31**	Week 31: PDSA cycle 1: Test definition of unreconciled medications and idea for tool with 1 registered nurse (RN) and 1 patient. Nurse on IDT was also a member of the valley team.
**32**	Week 32: PDSA cycle 2: Test addition of phrase “medication reconciliation & evaluation:” to SOC note template in EHR with 1 RN and 1 patient
**34**	Report association between unreconciled medications and hospitalization for data collected between approximately week 26 and 34 (see Table 4 and Figure 3)
**35**	Week 35: Home healthcare line adopted “medications visualized reviewed/reconciled” without realizing the IDT was testing a different phrase for SOC notes
**36**	Week 36: PDSA cycle 3: Pilot sheet of definitions for tool on how to handle medication reconciliation situations. Expand phrase to 2^nd^ field nurse on valley team and 2 triage nurses
**37**	Week 37: PDSA cycle 4: Test revised tool with 1 RN
**41**	Week 41: PDSA cycle 5: Revise and expand phrase “medication reconciliation & evaluation:” Adopted “medication reconciliation and evaluation:” as EHR template agency wide.
**44**	Week 44: PDSA cycle 6: Pharmacist/nurse joint home visit to refine tool
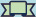	**Tests of change scaled to 1 geographic care team (valley team ∼ 1/5 of agency clinicians)**
**45**	Week 45: PDSA cycle 7 expanded use of revised tool; gave 1.5-hour in-service on scenarios 3, 5, 10-13 to practice using tool
**49**	Week 49: PDSA cycle 8: training scenario 6
**50**	Week 50: PDSA cycle 9: training scenario 7 & 8
**52**	Week 52: PDSA cycle 10: training scenario 1 & 2
**53**	Week 53: PDSA cycle 11: repeat modified training scenario 6 add 12
**55**	Week 55: PDSA cycle 12: Valley team training scenario 4; add tip sheet to summarize
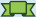	**Test of change scaled to all 5 geographic care teams**
**56**	Week 56: PSDA cycle 13: Roll out medication reconciliation tool to whole agency during 1-hour training session using a playground EHR environment (1 team per day)
**57**	Week 57: PDSA cycle 14: Medication evaluation training (trained RNs and therapists on separate days); added in basket reminder to reconcile medications for high-risk patients with 2 or more patient-reported meds appearing when screened by pharmacy.
**60**	Week 60: PDSA cycle 15: absentee training #1
**60-61**	Week 60-61: PSDA cycle 16: reconciliation scenario at clinician skills fair; evaluation of balancing measures.
**63**	Week 63: PDSA cycle 17: absentee training #2 / Manager reminder to clinicians / sharing progress /impact with agency
**66**	**End of individual patient level data collection; continue aggregate 30-day rehospitalization data collection in SHP**
**127**	**End 30-day rehospitalization data collection in SHP**

**Figure 2.**
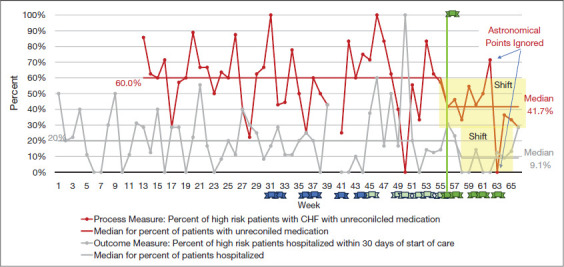
Home Health Patients with Heart Failure at High Risk for Hospitalization.

∗Color of flags denotes weeks with PDSA cycles on Figures 2 and 4 as well as S3-S6.

Abbreviations: EHR, electronic health record; IDT, interdisciplinary team; PDSA, Plan Do Study Act; RN, registered nurse; SHP, Strategic Healthcare Programs; SOC, start of care.

In Week 56, all the clinicians at the agency were trained. This included retraining the valley team members with the final version of the reconciliation tool and scenarios. See Table 3 for the text of the final tool. In Week 57, a pharmacist delivered a 1-hour training session for all agency clinicians on medication evaluation. The learning outcomes for this training are shown in Supplemental Table S3 at http://links.lww.com/HHN/A173.

**Table 3.** Medication Reconciliation Tool

	Adherent: Taking as Intended by Prescriber / OTC label		Prescriber intention Unknown	Non-adherent: Not Taking OR Not Taking as intended by prescriber/ OTC label		
	Taking as listed in EHR	Taking different than most recent order in EHR	Taking a med not listed as continue, stopped, or changed upon discharge / most recent med list	Taking differently than intended by prescriber	Taking medication that was intended to be stopped	Not taking
Current prescribed order/ OTC med/ supplement (may be routine or taken as needed)	Action: Add to Plan of Care (POC) list	Action^∗∗^: discontinue prior order^∗^ OR & Add new order to POC list		Action^∗∗^: Place on POC list as originally prescribed add comment OR *Discuss with patient & prescriber; determine best action.*		Action^∗∗^: Add/keep on POC list & add comment to order
Not prescribed			Action: *Clarify with prescriber* (e.g., hospital, nursing home, or provider may not have been aware patient was taking)		*Tell patient to stop med. If willing* Action∗∗: Remove from POC list & mark “not taking” OR discontinue order	Action∗∗: Remove from POC list & OR discontinue order

Abbreviations: EHR, electronic health record; med, medication; OTC, over the counter; POC, plan of care.

In Weeks 57 to 66, the pharmacy team altered its normal clinical service process to add a message to clinicians through the EHR to remind them to reconcile medications for patients who were screened by the pharmacy team and identified as high-risk and had two or more patient-reported unreconciled medications still not reconciled after SOC.

During Weeks 60 and 61, a skills fair for the whole agency had been planned separate from the project. The skills fair was used to confirm whether or not the initial training was attended and to reinforce the training. For those who missed the initial training absentee training was conducted in Weeks 60 and 63. The balancing measure poll regarding time for the new process was also conducted during the skills fair.

For comparison of the 30-day hospitalization rate, the baseline period was defined as Week 1 through the roll out of the intervention to the whole agency in Week 56, which occurred toward the end of August of 2019. Therefore, hospitalization data during the small-scale changes were included as baseline data. To mitigate the impact of the small-scale tests of change in assessing the baseline data, the IDT reviewed the 10 weeks immediately before and after the change as well as the same 10-week period for the previous year which occurred well before any small-scale test of change.

In line with the IHI training for QI projects, run charts were generated to evaluate trends, shifts, runs, and astronomical points using the statistical methods provided by IHI. The Healthcare Data Guide describes run chart interpretation with definitions of shifts, trends, too few or too many runs, and astronomical points (Provost & Murray, 2011).

## Results

The primary outcome, to reduce the 30-day hospitalization in high-risk patients with HF to less than 12% was met with the median percentage of patients with a 30-day hospitalization improving to 9.1%. The timing of the improvement in hospitalizations coincided with an improvement in reconciliation as measured by a change in the percentage of patients having one or more unreconciled medications per the IDT definitions (see Figure 2). Although both improvements were unlikely to have occurred by chance (*p* < .05), the improvement in hospitalization was not sustained beyond 8 weeks despite improvement in the medication reconciliation process measure.

In a post hoc analysis, the reduction of hospitalizations in patients with HF seemed to be driven by patients whose primary reason for HHC was not for HF. In this group, there was no discernable change in the reconciliation metric, but there was a significant improvement in hospitalizations (Figure S3 at http://links.lww.com/HHN/A178). Due to the small number of patients, it was not feasible to exclude SOCs done by clinicians who were not trained and still have meaningful weekly data.

**Figure 3.**
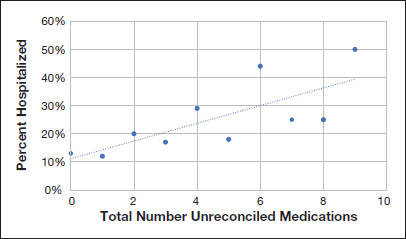
30-Day Hospitalization by # of Unreconciled Medications for Weeks 13 through 35.

**Figure 4.**
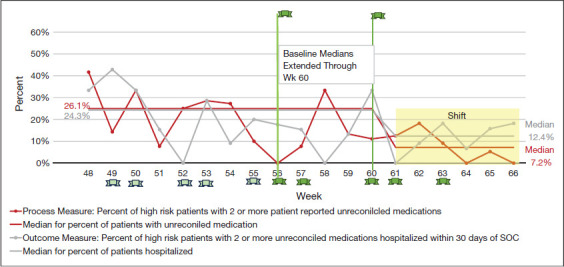
High Risk Patients with 2 or more patient reported unreconciled medications: Excludes patient admits by untrained clinicians & patients with missing reconciliation data.

There was an improvement of 23% in the baseline median for the process measure for secondary outcome measure 1, the percentage of all patients at high risk for hospitalization with at least one unreconciled medication (Figure S4 at http://links.lww.com/HHN/A179). Although no significant change was detected in 30-day hospitalization, significance was narrowly missed in Week 60. Based on baseline analysis showing increased hospitalizations among patients with two or more unreconciled medications, the analysis was further refined to focus on high-risk patients with two or more patient-reported unreconciled medications. (see Table 4 and Figure 3 for baseline data and Table 2 for project timeline placement) Patients admitted by clinicians who missed the training and/or lacked reconciliation data were excluded from this analysis (see Figure 4). This run chart shows significant improvements after Week 60 absentee training and the Week 61 skills fair. The improvement was sustained throughout the remainder of the manual data tracking portion of the measurement period and shows simultaneous improvements in the reconciliation process measure and hospitalizations outcome measure in all high-risk patients.

**Table 4.** Unreconciled Medications and Hospitalization in February & March 2019

	# patients hospitalized/# of patients	% hospitalized within 30 days of SOC	Mid *P* exact test (1-tail)
Any unreconciled medication	19/121	15.7%	
1 or 0 unreconciled medications	8/72	11.1%	*P* = .05239
2 or more unreconciled medications	11/49	22.4%	
Patient reported unreconciled medication			
1 or 0 unreconciled medications	13/105	12.4%	***P* =.01182**
2 or more unreconciled medications	6/16	37.5%	
Prescriber entered			
1 or 0 unreconciled medications	13/86	15.1%	*P* = .3861
2 or more unreconciled medications	6/35	17.1%	

Abbreviations: SOC = start of care 2 × 2 table calculated at https://www.openepi.com/TwobyTwo/TwobyTwo.htm

Tables 5, S4 at http://links.lww.com/HHN/A174, and S5 at http://links.lww.com/HHN/A175 complement Figures 2, S3, and S4 by describing the average percentage of patients hospitalized during different time points to allow for comparisons over similar sample sizes and time frames. Results for the percentage of patient with significant medication problems documented in the OASIS assessment question M2001 (Figures S5 at http://links.lww.com/HHN/A180) and number of discrepancies identified during a pharmacy home visit (Figure S6 at http://links.lww.com/HHN/A181) as articulated on the driver diagram are included in the supplement.

**Table 5.** 30-Day Hospitalization Average Before and After

Measure	Baseline Before Weeks 1-56		After full implementation Week 57-66		
All High-Risk Patients See Supplemental Figure S4	130/661	**19.7%**	18/125	**14.4%**	*p* = .08248
High-Risk Patients with CHF See Figure 2	91/431	**23.4%**	10/88	**11.4%**	***p*** = **.01516**
High-Risk Patients with Secondary Diagnosis of CHF; See Supplemental Figure S3	52/204	**25.5%**	3/45	**6.7%**	***p*** = **.001688**

Abbreviations: CHF (chronic heart failure) ***P*-value < .05 (bold)** 2 × 2 table calculated at https://www.openepi.com/TwobyTwo/TwobyTwo.htm

A total of 55 clinicians who had completed the medication reconciliation training responded to the balancing measure poll (Table 6).

**Table 6.** Balancing Measure

Time	*N* = 55
- No response	9%
- Less	24%
- Same	44%
- More	24%

**Figure 5.**
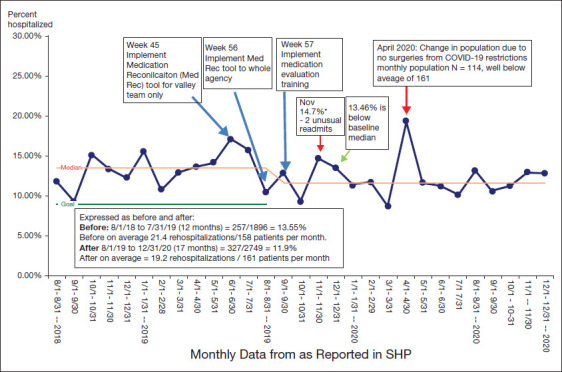
30-day Rehospitalizations for Whole Agency Hospital Discharge within 5 Days Prior to M0030 assessment at Start of Home Health.

Although it was not feasible to manually track the key process measures on reconciliation indefinitely, Figure 5 shows an improvement for secondary outcome measure 2, the percentage of patients with 30-day rehospitalizations for the whole agency as reported by Strategic Healthcare Programs (SHP). This occurred with an improvement in percentile rank (Figure S7 at http://links.lww.com/HHN/A182).

## Discussion

### Lessons

This QI project demonstrated that improving medication reconciliation and evaluation in high-risk home health patients, particularly in those with two or more unreconciled patient-reported medications led to a reduction in 30-day hospitalizations. Additionally, it is likely that this project contributed to an improvement in 30-day rehospitalizations, reducing their overall occurrence by an average of about two patients per month. This metric provides a global overview and external validity for the project from SHP data as the improvement occurred at the time of the intervention. Unreconciled medications are strongly correlated with increased 30-day hospitalizations at the project HHC agency. This is particularly true for patients with two or more unreconciled medications listed as patient-reported and identified in the EHR after the SOC visit. The improvement in percentile rank shows the decrease in hospitalizations was not simply part of a national trend showing a decrease in hospitalizations year over year, but rather an improvement relative to other HHC agencies. Coleman et al. proposed patient- and system-associated QI solutions to ensure patient safety (Coleman et al., 2005). The current project aligns with the results demonstrated by Coleman et al. that MRPs may lead to 30-day hospitalization and expands upon the results by providing a tool to ensure the agency's EHR's systems were used optimally to communicate medication reconciliation and evaluation issues (Coleman et al., 2005).

The baseline median of 60% of patients with HF having an unreconciled medication per the operational definition following admission to HHC approximated the 71.2% of HF patients with a medication reconciliation problem documented by Foust et al. among older adults with HF transitioning from hospital to home (Foust et al., 2012). In the Foust et al. study, 39.9% had two or more medication reconciliation problems (Foust et al., 2012). In the present project only 26.1% of high-risk patients had two or more unreconciled medications at baseline after the HHC clinician had done a reconciliation. Although the IDT's definitions of reconciliation are different from the aforementioned studies, there is value in seeing the similarities in results given the ubiquitous nature of medication reconciliation problems. Surprisingly, in developing the tool, 13 unique reconciliation scenarios were identified that required different steps for the clinicians to resolve. This underscores the complexity of reconciliation in this setting. The primary tool used at this HHC agency may have applicability to medication reconciliation in a multitude of practice settings, including hospitals and primary care. The tool is meant to be used with the patient's medications in front of the clinician during a patient/caregiver interview. The goal of the tool is to help clinicians consider whether a medication presented by the patient is currently prescribed/current medication for the patient and whether the patient is adherent to the prescriber's orders. The tool then guides the clinician to update the EHR based on that information. These actions could be customized to fit any EHR or practice setting. The tool is meant to consider all possibilities when presented with a medication that may or may not be listed in the EHR.

The balancing measure indicated most clinicians did not require more time for medication reconciliation and evaluation. The fact that some noted a decrease in time may indicate efficiencies from streamlining key processes. The collaborative nature of the project and gathering feedback from the clinicians may have helped to ensure this would be helpful rather than burdensome and utilizing pharmacist expertise may have helped to streamline the process.

The reduction in hospitalizations in the post hoc subgroup with secondary HF despite not experiencing improvement in reconciliation may be explained by improved medication evaluation or by having too small of number of patients each week to easily show a change on a run chart.

Regarding Figures 5 and S7, statistical significance was narrowly missed in November 2019. The IDT discussed two unusual readmissions that altered results in the final weeks of manually tracking the key process measures. Had those not occurred statistical significance would have been achieved in the immediate 6 months following the intervention. The decision was made to continue tracking the aggregate outcome to evaluate for sustained improvement. COVID-19 subsequently impacted the results of the next 6 months by greatly altering the population of patients who were admitted to the agency in April 2020. During this month, there was a greater proportion of high-risk patients and a lower number of post-acute surgery patients. Eight data points are presented after the major disruption in the population caused by COVID-19 that demonstrate a statistically significant improvement, albeit without any process metrics to show the connection to improved medication reconciliation and evaluation.

The improvement demonstrated with this project led to discussions regarding ways to spread this change to other HHC agencies within the larger organization. Additionally, several improvements to the EHR were also proposed to facilitate reconciliation and evaluation.

## Limitations

QI projects are based on having “just enough” data to make an improvement. They are not designed as a randomized controlled trial with the intention of generating new knowledge. The intervention was tailored to the specific needs of this site and was modified and developed throughout the project. As a result, there are some inherent limitations to its applicability to other HHC agencies or other settings.

Baseline data were impacted from Week 35 forward due to adoption of the prompt for SOC notes. The small-scale tests of change had a notable impact on hospitalizations starting with Week 37. As the IDT included baseline data through Week 56, these impacts may have biased the before and after data as well as the baseline medians toward not showing any benefit. Additionally, the project lacked a routine process measure to evaluate the impact of the medication evaluation training. Similarly, the links between the primary and secondary drivers were not 100% accurate. A different way to measure the impact of medication evaluation skills and connect them with hospitalization may have been helpful. External factors as well as the number of referrals to HHC from different referral sources may have affected hospitalizations. There is also the potential that pharmacy team interventions could have impacted outcomes, though there was no change to pharmacy team services during the QI period. On occasion the IDT was unable to capture the number of unreconciled medications because the patient was readmitted to the hospital before the chart review could be completed. Additionally, the process of manually capturing this information was not sustainable due to the time involved. Future projects may benefit from software support/development to improve data capture.

As reported by Reed et al., survey questions are often problematic in QI projects (Reed et al., 2023). The IDT could have further developed the poll question or have selected a different balancing measure.

Finally, the number of unreconciled medications may be a surrogate marker for a number of variables that could affect reconciliation other than training, such as challenges in receiving a medication list from different referral sources (hospitals, providers, skilled nursing facilities) outside the same health system rather than within the same system as well as time of admission (weekends vs weekdays). There was no control for these factors. No control was done for patient's overall health status.

## Conclusion

A collaborative, pharmacist-led project to improve medication reconciliation and evaluation in HHC resulted in a significant reduction in hospitalizations in high-risk patients with HF and was temporally associated with a sustained reduction in rehospitalization in the overall HHC population, though there were two outlying situations. Identifying and addressing challenges HHC clinicians face in medication reconciliation and evaluation and consistently reinforcing this topic may improve outcomes. Involving pharmacists to make this an interdisciplinary process may help due to the complex nature of medication regimens encountered in HHC and the impact that medication use can have on quality measures. Further work is needed to determine the sustainability of this intervention and whether these findings are applicable to other HHC agencies.
